# Thaumatin-Like Protein (Pru av 2) Is a Cherry Allergen That Triggers Percutaneous Sensitization in Mice

**DOI:** 10.3390/foods10010134

**Published:** 2021-01-10

**Authors:** Eri Izumi, Shota Hidaka, Ayako Hiroi, Serina Kinugasa, Erika Yano, Nobuhiro Zaima, Tatsuya Moriyama

**Affiliations:** 1 Department of Applied Biological Chemistry, Graduate School of Agriculture, Kindai University, 3327-204 Naka-machi, Nara 631-8505, Japan; erizumi.123@gmail.com (E.I.); shotahidaka14@gmail.com (S.H.); ayako.hiroi.0710@gmail.com (A.H.); serina.kinugasa0106@gmail.com (S.K.); pyonchangoods@yahoo.co.jp (E.Y.); zaima@nara.kindai.ac.jp (N.Z.); 2 Agricultural Technology and Innovation Research Institute, Kindai University, 3327-204 Naka-machi, Nara 631-8505, Japan

**Keywords:** cherry allergens, thaumatin-like protein, Pru av 2, allergenicity, percutaneous sensitization

## Abstract

Numerous recent studies have suggested that food allergens enter the skin and predispose individuals to food allergies through the production of IgE antibodies in the body. Cherries are a popular fruit eaten worldwide. However, cherries are an allergenic food and percutaneous sensitization with cherry allergens through the perioral region may occur while ingesting cherries. The identity of the cherry protein that triggers percutaneous sensitization in humans or animal models remains unknown. In this study, the backs of BALB/c mice were shaved and crude cherry extracts containing sodium dodecyl sulfate were applied to the skin. Thereafter, the cherry-specific IgE and IgG1 antibodies generated and secreted in response to the epidermal application were measured using an enzyme-linked immunosorbent assay or immunoblotting. Skin exposure to cherry extracts elevated cherry-specific IgG1 levels. Application of fractionated and purified cherry proteins (antigen candidates for percutaneous sensitization) that bound to the IgG1 antibodies led to the identification of a thaumatin-like protein (Pru av 2). This molecule is known as the major cherry allergen that affects humans. In conclusion, our study identified Pru av 2 as a cherry allergen that triggers percutaneous sensitization in mice for the first time.

## 1. Introduction

Food allergies are a growing public health problem that compromises the quality of life. An estimated 5% of adults and 8% of children suffer from a food allergy [1]. Typically, the ingested food is digested and absorbed without causing immune reactions that adversely affect the body. This function, called oral tolerance, prevents excessive immune responses to innocuous food proteins [2]. However, if the immune tolerance is compromised by any factor, a T helper (Th) 2 type of immune response is triggered, leading to developing a food allergy through the production or response of IgE antibodies. Development of a food allergy is initiated by sensitization, in which IgE-specific antibodies are produced for an antigenic protein entering the body and bind to mast cells and basophils [3].

Recently, the cases of a wheat allergy developed in users of soaps containing hydrolyzed wheat reported in Japan have attracted attention to a new sensitization route—called percutaneous sensitization. Here, it is believed that hydrolyzed wheat entered through the percutaneous and transmucosal routes, rather than through the oral route, and sensitization was established [4,5,6]. Several cases on percutaneous sensitizations have been reported [7,8], including cases of soy allergy after the use of soy-based cosmetics and peanut allergy after the use of peanut oil [9,10].

Owing to the relationship between oral tolerance and percutaneous sensitization, the dual-antigen exposure hypothesis has been proposed [8]. It is hypothesized that ingestion of food leads to immune tolerance and prevents a food allergy, whereas the penetration of antigens through the skin and mucosa without passing through the intestinal tract induces IgE production, leading to a food allergy. Food proteins that adhere to the skin or become airborne are taken up by Langerhans cells on the skin, leading to a predominantly Th2-type immune response and production of cytokines such as interleukin (IL)-4, -5, and -13. Finally, the binding of the B cell-produced IgE to mast cells establishes immune sensitization. Conversely, food entering the intestine elicits a predominantly Th1-type immune response, wherein regulatory T cells (Tregs) form and suppress the Th2-type immune response. Therefore, a food allergy may be prevented if the allergen is ingested and immune tolerance is established before it enters through the percutaneous route. Indeed, early consumption of peanuts reportedly leads to oral immune tolerance and suppresses the progression of the peanut allergy [11].

Damage to the barrier function of the skin and inflammation are factors causing percutaneous sensitization [12,13]. In these processes, the participation of thymic stromal lymphopoietin (TSLP) produced from epithelial cells has been reported [14,15]; however, studies on what food proteins trigger percutaneous sensitization are scarce. Previously, we identified Gly m 5 and Gly m 6, known as principal soybean allergens, as the triggers of percutaneous sensitization using a mouse model [16]. Using the same model, soybean trypsin inhibitor (Gly m TI) was also identified as a soybean allergen that causes percutaneous sensitization [17]. These food allergens are also the primary soybean allergens in humans, suggesting this mouse model might be useful for the identification of allergens that cause percutaneous sensitization in humans. Here, we extend the application of this model to identify such antigens in fruits.

The cherry is a member of the Rosaceae family. Although allergies to cherries are not common, several cases have been reported [18,19]. Principal cherry allergens [20] include Pru av 1 (PR-10), Pru av 2 (thaumatin-like proteins), Pru av 3 (nsLTP), and Pru av 4 (profilin). Moreover, these can induce cross-reactivity with other vegetables or fruits, including peach allergens Pru p 3 and Pru p 2, which are highly similar to Pru av 3 and Pru av 2, respectively. In addition, some allergies caused by cherries are often associated with birch pollinosis. This is due to the cross-reactivity between birch pollen antigens and cherry proteins. Recently, cherries have been used in cosmetics such as lip care products, which would present opportunities for percutaneous sensitization. Therefore, cherries were used as one of the fruit foods that could cause percutaneously sensitization.

In this study, we applied cherry extracts on mouse dorsal skin to investigate whether cherry proteins can induce percutaneous sensitization. Furthermore, we investigated the primary cherry allergens responsible for percutaneous sensitization by identifying IgE- and IgG1-binding cherry proteins produced in mice by means of immunoblotting targeting for all molecular weight areas.

## 2. Materials and Methods

### 2.1. Materials

Enzyme-linked immunosorbent assay (ELISA) plates were purchased from AGC (Tokyo, Japan). Horseradish-peroxidase (HRP)-conjugated goat antimouse IgE antibody was purchased from Southern Biotech (Birmingham, AL, USA). HRP-conjugated goat antimouse IgG1-antibody was purchased from Bethyl Laboratories (Montgomery, TX, USA). 3,3′,5,5′-tetramethylbenzidine (TMB) peroxidase substrate was purchased from Kirkegaard & Perry Laboratories (Gaithersburg, MD, USA). Coomassie Brilliant Blue R-250, Bradford protein assay reagent, and pentobarbital sodium salt were purchased from Nacalai Tesque (Kyoto, Japan). Enhanced chemiluminescence (ECL) Western blotting substrate and X-ray films (Amersham Hyperfilm MP) were purchased from GE Healthcare (Chalfont St. Giles, UK). Immunoreactive enhancers, CanGet Signal solutions 1 and 2, were purchased from TOYOBO (Osaka, Japan). All other chemicals used in this study were of the highest purity available.

### 2.2. Preparation of Cherry Extract and Protein Estimation

American (sweet) cherries (cherry: *Prunus avium*) were purchased locally. Cherry seeds were removed, and the edible portion was homogenized using a commercial food mixer and passed through four layers of gauze. The extract was centrifuged at 10,000× *g* for 10 min at 4 °C, and the supernatant was collected. The protein levels in the cherry extracts were determined using the Bradford method [21], using γ-globulin as the standard (Bio-Rad, Hercules, CA, USA) and Protein Assay Coomassie brilliant blue (CBB) solution (Nacalai Tesque), and the absorbance was measured at 595 nm.

### 2.3. Animal Studies

**Figure 1:**
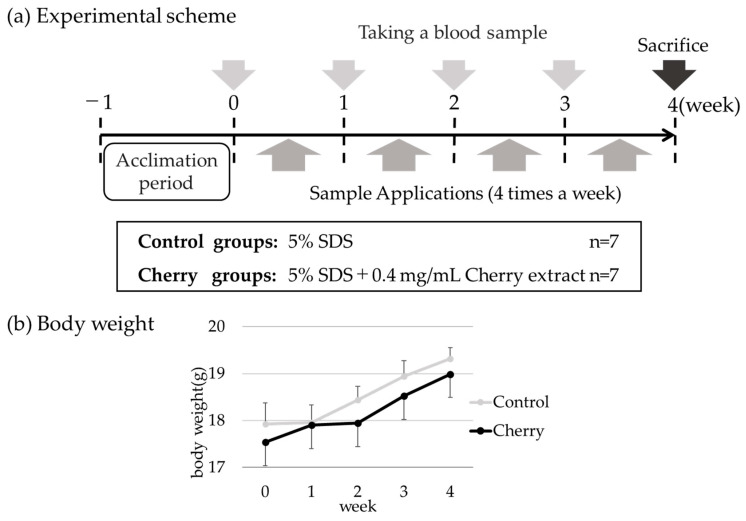
Percutaneous sensitization with cherry extract does not affect mice growth. (**a**) Schema of the percutaneous sensitization protocol. Detailed information is described in “Materials and methods.” (**b**) Body weights of the control group and cherry group. Data are presented as means ± standard deviations (SD); control group (*n* = 7), cherry group (*n* = 7).

Six-week-old female BALB/c mice were purchased from Japan SLCs (Shizuoka, Japan) and used for percutaneous sensitization treatment, as shown in Figure 1a. Mice were fed commercial chow (MF, Oriental Yeast, Tokyo, Japan) and water was provided ad libitum. The commercial chow did not contain cherry proteins. Mice were acclimated for 7 days before the start of the experiment. All animal experiments were approved by the Kindai University Animal Experiment Commission (Approval No. KAAG-26-004).

### 2.4. Induction of Percutaneous Sensitization

After shaving the occipital region of the mice with an electric shaver under anesthetic conditions, the remaining hair was removed with hair removal cream (Veet, Reckitt Denckiser, Berkshire, UK), and tape stripping was performed 10 times. A mixture of midazolam (Astellas Pharma, Tokyo, Japan), butorphanol (Meiji Seika Pharma, Tokyo, Japan), and medetomidine (Nippon Zenyaku Kogyo, Fukushima, Japan) was used for anesthesia during shaving and the following treatments. This shaving treatment was performed weekly. Percutaneous sensitization was induced by applying the respective samples to the treated skin. Mice were divided into either the control or cherry groups (*n* = 7 in each group).

The control group received only 5% sodium dodecyl sulfate (SDS), and the cherry group received cherry extract (0.4 mg protein/mL) in 5% SDS. The sample (50 μL) was applied to the epidermis using a micropipette four times a week for 4 weeks. Serum samples were collected weekly. At 4 weeks, all mice were anesthetized with sodium pentobarbital salt by an intraperitoneal injection. Subsequently, the mice were sacrificed by cervical dislocation. Mice were weighed weekly to determine if the growth of the mice was not inhibited. The animal experimental schema is summarized in Figure 1a.

### 2.5. Enzyme-Linked Immunosorbent Assay (ELISA)

ELISA was used to determine the binding of murine sera IgE and IgG1, which are Th2-related immunoglobulins in mice, to cherry proteins. Samples (50 μL of 20 μg/mL) were prepared in PBS (137 mM NaCl, 2.7 mM KCl, 10 mM Na_2_HPO_4_, 1.76 mM KH_2_PO_4_; pH 7.4) and solidified in an ELISA plate (AGC) at 4 °C overnight. Plates were blocked at room temperature for 1 h using 100 μL 1% BSA prepared in PBST (137 mM NaCl, 2.7 mM KCl, 10 mM Na_2_HPO_4_, 1.76 mM K H_2_PO_4_, 0.1% Tween-20; pH 7.4) and washed three times with 100 μL PBST. Subsequently, 50 μL each of the diluted primary antibodies (mouse serum) was added, incubated at 37 °C for 1 h, and washed five times with 100 μL PBST. To determine IgE levels, each serum was diluted 20-fold with PBST containing 1% BSA. To determine IgG1 levels, each serum was diluted 20-fold with CanGet Signal soln1 (TOYOBO). Next, 50 μL each of the diluted secondary antibodies was added and incubated at 37 °C for 1 h. 8000-fold diluted rat monoclonal 23G3 antimouse IgE epsilon chain HRP-conjugated secondary antibodies (Abcam, Cambridge, UK) prepared in PBST containing 1% BSA was used to measure IgE levels, and 50,000-fold diluted goat antimouse IgG1 antibody HRP-conjugated secondary antibodies (Bethyl Laboratories: Montgomery, TX, USA) diluted in CanGet Signal soln2 (TOYOBO) was used to measure IgG1 levels. After washing five times with 100 μL PBST, 50 μL of TMB (Kirkegaard & Perry Laboratories) was added to detect the bound secondary antibody. Finally, 50 μL 1 M phosphoric acid was added to stop the reactions. The absorbance at 450 nm was measured using a plate reader (Wallac Arvo SX 1420 Multilabel Counter; PerkinElmer, Waltham, MA, USA). Measurements were collected using two wells, and the means were used for statistical analysis.

### 2.6. Electrophoresis and Immunoblotting

Proteins (approximately 20 μg/lane) contained in cherry extracts were separated for 30 min (constant 200 V) using SDS with polyacrylamide gel electrophoresis (SDS-PAGE) [22]. For confirmation of protein patterns, 12.5% SDS-PAGE gels were stained with CBB.

For immunoblotting, the gels were transferred to polyvinylidene difluoride (PVDF) membranes (Immobilon-P; Millipore, Burlington, MA, USA) for 30 min (constant 15 V) after SDS-PAGE with the semidry blotting method [23]. Blocking was performed by immersing the PVDF membrane in 5% skim milk in PBST for 1 h. For detection of IgE bindings, the PVDF membranes were washed twice with PBST for 5 min and incubated overnight at 4 °C with the serum diluted 20-fold in the CanGet Signal soln1 (TOYOBO). IgG1 bindings were similarly detected by incubating the membrane with the serum diluted 20-fold in the CanGet Signal soln1. After three 10-min washes with PBST, IgE levels were measured using HRP-conjugated antimouse IgE antibody diluted 8000-fold in the 5% skim milk in PBST, which reacted for 1 h. IgG1 levels were measured with HRP-conjugated goat antimouse IgG1 antibody diluted 50,000-fold in the skim milk solution and reacted under similar conditions. Membranes were washed four times with PBST for 10 min each and incubated for 1 min with Amersham ECL Western Blotting Detection Reagents (GE Healthcare). Luminescence signals were detected with X-ray films (Amersham Hyperfilm MP; GE Healthcare) for 10–30 min.

### 2.7. Purification and Identification of IgG1-Reactive Major Cherry Proteins

The major IgG1-binding protein (27 kDa) was purified via ammonium sulfate precipitation, followed with ion-exchange chromatography and gel-filtration chromatography to identify percutaneous sensitizing antigens in cherry extracts. SDS-PAGE and immunoblotting were performed after each step. IgG1-binding proteins were detected using mouse serum IgG1 by immunoblotting. Mouse serum samples were prepared by mixing equal amounts of sera from five cherry-treated mice collected at 4 weeks and used as primary antibodies for immunoblotting.

#### 2.7.1. Ammonium Sulfate Precipitation

Cherry extract (100 mL) in buffer A (10 mM Tris-HCl, pH 7.5, 1 mM EDTA) containing the protease inhibitor mix (Nacalai Tesque) was freshly prepared for purification. Ammonium sulfate was added stepwise to the above solution, and a precipitate was obtained by centrifugation at each step. Samples precipitated with ammonium sulfate (0–20%, 20–40%, 40–60%, and 60–80%) were recovered and resolved with 10 mL of buffer A.

#### 2.7.2. Ion-Exchange Chromatography

Samples obtained after ammonium sulfate precipitation were desalted using a PD-10 mini-column (GE Healthcare), followed by a manually prepared ion-exchange chromatography using Super Q anion-exchange gels (TOSOH, Tokyo, Japan) (bed volume: 5 mL) to separate proteins by a stepwise method (0 M, 0.05 M, 0.1 M, 0.2 M, 0.3 M, 0.4 M, 0.5 M, and 1 M of NaCl).

#### 2.7.3. Gel-Filtration Chromatography

Samples obtained from ion-exchange chromatography were concentrated using Amicon Ultra-15 10K filters (Millipore) and subjected to gel-filtration chromatography (TOSOH G3000SW) using high performance liquid chromatography (HPLC; Hitachi L-6200) for further purification (flow rate: 0.5 mL/min). Elution fractions (0.5 mL/fraction) from gel-filtration chromatography were used to perform CBB staining and immunoblotting after resolving the proteins via SDS-PAGE as described above.

### 2.8. N-Terminal Amino Acid Sequence Analysis

The sample obtained from gel-filtration chromatography was subjected to SDS-PAGE and transferred onto a PVDF membrane as described above. The PVDF membrane was stained with Ponceau S and the band corresponding to IgG1-binding protein was cut and collected. The N-terminal amino acid sequence of the sensitizing antigen was determined using Edman degradation [24] (analyzed at Hokkaido System Science, Sapporo, Japan).

### 2.9. Statistical Analysis

The two-tailed Student’s *t*-test was used for comparing more than one group. A significant difference was defined when the *p*-value was < 0.05 (* *p <* 0.05, significantly different).

## 3. Results

### 3.1. Percutaneous Sensitization with Cherry Extract Did Not Affect Mouse Growth

Six-week-old female BALB/c mice were acclimated for 1 week before starting the percutaneous sensitization treatment. Cherry extracts (0.4 mg/mL with 5% SDS) were applied to the cherry group, and SDS (5%) was applied to the control group (Figure 1a). Body weights were measured weekly to ensure that the mice were not under excessive stress by the percutaneous application treatment. The body weights of mice gradually increased with no differences between the groups suggesting normal growth in both groups (Figure 1b).

### 3.2. Changes in Cherry-Specific IgE and IgG1 Antibodies Determined Using ELISA

**Figure 2:**
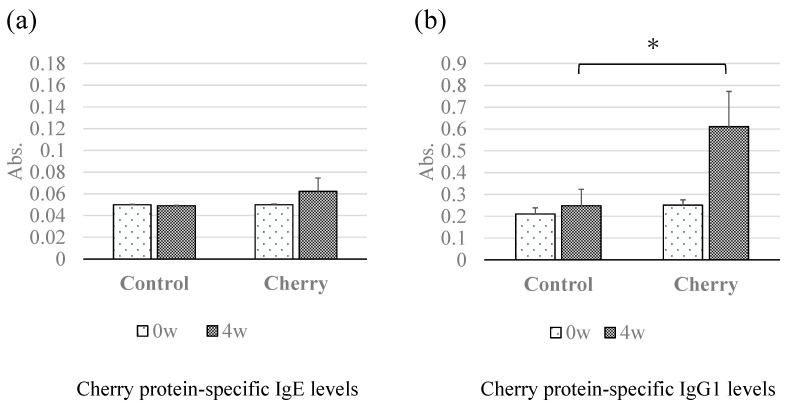
Effect of percutaneous sensitization with cherry extracts on cherry-specific antibody levels in mice sera. Cherry-protein-specific IgE levels (**a**) and IgG1 levels (**b**) in control and cherry groups at 0 and 4 weeks were determined using ELISA performed with cherry protein-coated plates. The ELISA data is presented in absorbance values (Abs). Absorbance data are presented as means ± standard deviations (SD); control group (*n* = 7), cherry group (*n* = 7). * *p <* 0.05.

We sought to examine whether levels of cherry-protein-specific serum IgE and IgG1 changed in response to percutaneous sensitization. Mouse sera collected at 0 and 4 weeks after percutaneous sensitization treatments were used for IgE- and IgG1-ELISA. In the sera collected at week 4, whereas the IgE levels in the cherry-exposed group were higher than those in the control group were, no significant differences in cherry-protein-specific IgE levels between the two groups (Figure 2a) were noted. Remarkably, cherry-protein-specific IgG1 levels were significantly higher in sera collected at 4 weeks from the cherry group compared with the control group (Figure 2b).

### 3.3. Detection of IgE- and IgG1-Binding Cherry Proteins Using Immunoblotting

**Figure 3:**
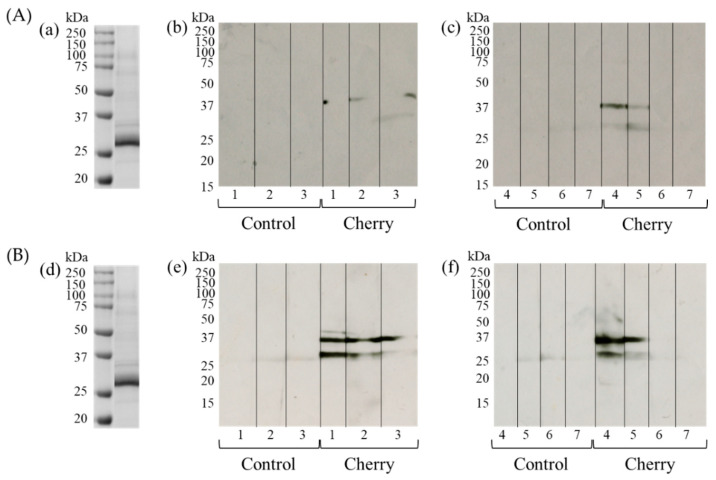
Detection of IgE-binding (**A**) and IgG1-binding (**B**) cherry proteins using immunoblotting. (**A**) (**a**) Coomassie brilliant blue staining of cherry proteins. (**b**,**c**) Immunoblotting using cherry proteins, individual mice sera, and HRP-labeled antimouse IgE antibody. The numbers indicate the individual mice in control and cherry groups. Molecular mass was standed for kDa. (**B**) (**d**) Coomassie brilliant blue staining of cherry proteins. (**e**,**f**) Immunoblotting using cherry proteins, individual mice sera, and HRP-labeled antimouse IgG1 antibody. The numbers indicate the individual mice in control and cherry groups. No specific bands were detected higher and lower areas than 15–250 kDa areas in sera from mice in both groups (data not shown).

Next, we examined the molecular weights of the sensitizing cherry antigens using immunoblotting. The crude cherry extract was prepared for SDS-PAGE and transferred to PVDF. Immunoblotting using anti-IgE antibodies and sera from multiple cherry-exposed mice detected specific IgE-binding protein bands in the cherry extract at approximately 27 kDa and 35 kDa (Figure 3A). We found no antibody reactive bands in higher and lower areas than 15–250 kDa areas (data not shown). No specific bands were detected in sera from mice in the control group. Similarly, specific IgG1-binding bands at approximately 27 kDa and 35 kDa were detected in some individual mice of the cherry group (Figure 3B). Together, these results suggest that IgE and IgG1 antibodies produced and secreted into sera could bind specific cherry proteins.

### 3.4. Identification of Cherry Antigens That Trigger Percutaneous Sensitization

As the cherry protein bands (approximately 27 kDa and 35 kDa) that bind IgE and IgG1 were considered the same, the more sensitive IgG1 was used for the subsequent purification and identification of the sensitizing antigens.

**Figure 4:**
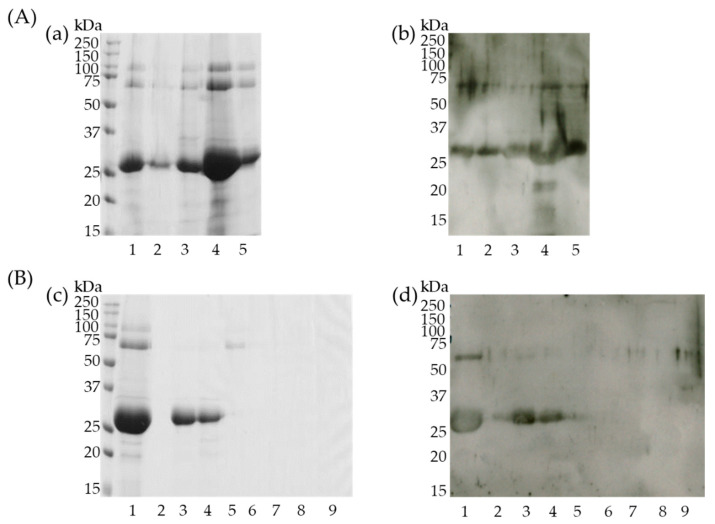
Fractionation of cherry proteins using ammonium sulfate precipitation (**A**) and ion-exchange chromatography (**B**). (**a**,**c**) Coomassie brilliant blue staining of fractionated cherry proteins. (**b**,**d**) Immunoblotting using fractionated cherry proteins, mixed mice sera, and HRP-labeled antimouse IgG1 antibody. (**A**) Lane 1, crude extracted sample; lanes 2~5, fractionated samples with 0–20% (2), 20–40% (3), 40–60% (4) and 60–80% (5) of ammonium sulfate precipitations. (**B**) Lane 1, fractionated sample with 40–60% ammonium sulfate precipitation; lane 2, flow-through fraction from ion-exchange chromatography; lanes 3~9, sample eluted with buffer A containing 0.05 M (3), 0.1 M (4), 0.2 M (5), 0.3 M (6), 0.4 M (7), 0.5 M (8), and 1 M (9) of NaCl.

The cherry extract was first fractionated using ammonium sulfate. CBB staining (Figure 4A-a) and immunoblotting (Figure 4A-b) of each sample after ammonium sulfate precipitation identified that the 27 kDa IgG1-binding protein band was most abundant in the 40–60% ammonium sulfate precipitated samples. Subsequently, this fraction was used for ion-exchange chromatography.

CBB staining (Figure 4B-c) and immunoblotting (Figure 4B-d) of ion-exchange chromatography fractions revealed that the ~27 kDa protein was present in the fraction eluted with buffer containing 0.05 M NaCl. This fraction was used for the subsequent gel-filtration chromatography.

**Figure 5:**
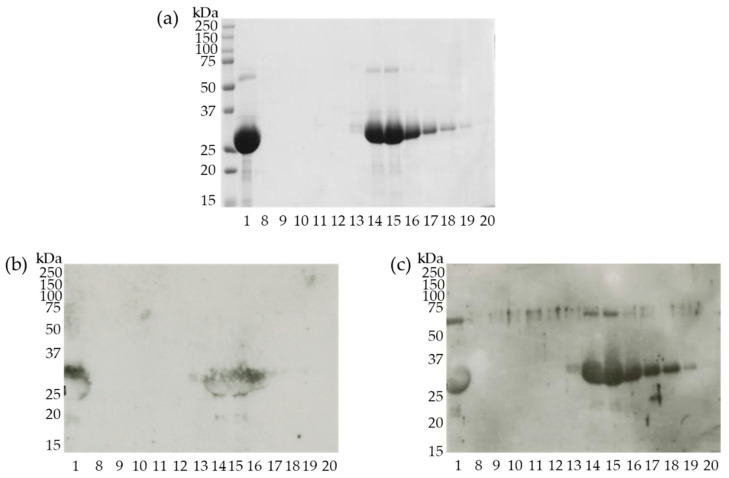
Separation of cherry proteins using gel-filtration HPLC (**a**) Coomassie brilliant blue staining of separated cherry proteins. (**b**) Immunoblotting using fractionated cherry proteins, mice sera, and HRP-labeled antimouse IgE antibody. (**c**) Immunoblotting using fractionated cherry proteins, mice sera, and HRP-labeled antimouse IgG1 antibody. 1, applied sample; 8–20, corresponding eluted faction number.

Fractions (No. 8 to 20) from the gel-filtration HPLC chromatography that contained proteins (confirmed by A_280_ nm detection) were analyzed using CBB staining and immunoblotting. Fraction No. 15 contained the highest purity and concentration of the 27 kDa protein (Figure 5a,c) was transferred to a PVDF membrane and the N-terminal amino acid sequence of this protein was identified using Edman degradation.

**Figure 6:**
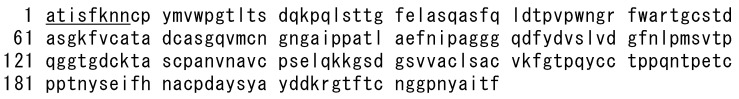
Amino acid sequence of Pru av 2. The N-terminal amino acid residues identified via Edman degradation are underlined. (GenBank Protein accession No. AAB38064).

The N-terminal amino acid sequence of the 27 kDa protein was “ATISFKNN” (Table 1). A homology search using BLAST for proteins with matched N-terminal sequences was performed, and the sequence was consistent with Pru av 2 (thaumatin-like protein) cherry protein (Figure 6).

### 3.5. Semi-Purified 27 kDa Protein Binds IgE

To verify IgE binding to the partially purified 27 kDa protein, all the fractions eluted from the gel-filtration column were subjected to immunoblotting using pooled mice sera collected at 4 weeks from the cherry group. As shown in Figure 5b, IgE could bind the corresponding protein bands similar to IgG1 (Figure 5c), suggesting that the 27 kDa protein could be recognized and bind to both IgE and IgG1 antibodies produced in mice that were percutaneously treated with cherry extracts.

## 4. Discussion

In this study, we applied cherry extract (0.4 mg/mL) containing 5% SDS to the shaved dorsal skin of BALB/c mice for 4 weeks to investigate whether cherry proteins can cause percutaneous sensitization. We also investigated the percutaneous sensitizing cherry antigen. During sample application, the mouse skin was disrupted by the adhesive tape. Moreover, the addition of SDS, a surfactant, to the samples likely promoted destruction of the skin barrier. Application of the cherry extract following skin barrier disruption elicited the production of cherry-protein-specific IgE and IgG1 antibodies, which were detectable in sera. Thus, we demonstrated, for the first time, the ability of cherry proteins to mediate percutaneous sensitization in a mouse model.

In this study, we wanted to use relative severe conditions; thus, we used SDS to mimic the severely damaged skin—for example, atopic dermatitis. In addition, we tried to mimic the condition of addition of cherry to soap and cosmetics applied on damaged skin by using SDS. In the future, it should be investigated whether cherry proteins could percutaneously sensitize without SDS for milder conditions.

While cherry-specific IgE levels were not significantly different between the control and the cherry group (Figure 2a), significant differences were observed in cherry-specific IgG1 levels (Figure 2b). Immunoblotting detected both IgE- and IgG1-binding protein bands at approximately 27 kDa and 35 kDa (Figure 3). These results suggest that the cherry protein possesses a measurable percutaneous sensitization potential in this mouse model system. We speculate these two proteins are potential candidates for percutaneous sensitizing cherry antigens. In our previous study, we observed the significant elevation of specific IgE and IgG1 using soybean proteins. The differences in the antibody production levels might be caused by the differences in the applied protein concentrations (50 mg/mL of soybean protein vs. 0.4 mg/mL cherry protein) [16]. In addition, the differences might be caused by the differences in the sensitization powers of the responsible antigens.

The ~27 kDa band was more densely stained with CBB than the ~35 kDa band, suggesting that it was more abundant. However, the ~35 kDa band was stained more densely in the immunoblot, suggesting that more potent and/or abundant antibodies against this ~35 kDa band protein might be produced. This suggests that cherry contains more IgE- and IgG1-binding proteins around 27 kDa, although the percutaneous sensitizing potential of the ~35 kDa protein might be stronger than the ~27 kDa protein. We first attempted to purify both the 27 kDa and 35 kDa allergen candidates; however, the 35 kDa protein band could not be detected by immunoblotting during the purification steps. We speculate that the antibody-binding properties of the protein might have been lost during the purification steps. In the future, modifications to the experimental method are required to facilitate the identification of the 35 kDa cherry protein that triggers percutaneous sensitization.

N-terminal amino acid sequence analysis of the 27 kDa protein detected by immunoblotting revealed this to be Pru av 2 (thaumatin-like protein), a panallergen in cherries that affects humans (Table 1 and Figure 6).

Thaumatin-like proteins (TLPs) are polypeptides of approximately 200 amino acid residues that share sequence similarity with a sweet-tasting protein, thaumatin [25,26]. Several members of the plant TLP superfamily found in fruits and conifers have been reported to act as food and pollen allergens, respectively [27]. TLP allergens are found in cherries (*Prunus avium* L; Pru av 2) [28], kiwis (*Actinidia chinensis* Planch; Act c 2) [29], apples (*Malus domestica*; Mal d 2) [30], and bananas (*Musa acuminate* Colla) [31].

As expression levels of TLPs are induced by stresses such as an attack from a pathogen or pest, plant TLPs are classified as the pathogenesis-related (PR) protein family 5 (PR5), one of the 17 families of defense-related PR proteins [32,33]. Although how this protein could act as a defensive molecule is still unknown, the antifungal activity of this protein has been reported [26]. Several TLPs exhibit binding to water-insoluble β-1,3-glycans [34,35,36], which are a common component of the fungal cell wall. Typically, TLPs contain 16 conserved cysteine residues [26]. The disulfide bonds formed by these conserved cysteines help stabilize the molecule and allow for correct folding and high stability under extreme thermal and pH conditions [37]. In addition, this protein also exerts resistance to protease degradation [38]. We observed that cherry TLP (Pru av 2) was not digested by pepsin or trypsin/chymotrypsin in vitro (data not shown). These properties suggest that TLPs have the potential for evoking allergic systemic responses and/or oral allergy syndrome after oral administration in individuals percutaneously sensitized with this allergen.

TLPs are also known as one of the pollen antigens responsible for certain types of pollinosis. Of these, Jun a 3 and Cry j 3 are found in the mountain cedar (*Juniperus ashei* J. Buchholz) [39] and the Japanese cedar (*Cryptomeria japonica* L. f.), respectively [40]. In addition, TLPs were reported to be responsible for cross-reactivity between fruit and pollen [41].

Thus, TLPs may sensitize through enteral, percutaneous, and airborne routes. In addition, because Pru av 2 is a major human allergen in cherries, the mouse model used here mimics the mechanism of (percutaneous) sensitization in humans, suggesting this mouse model may be applicable to search for unknown allergens. A relevant follow-up experiment would be to determine whether sensitization can be mitigated with cherry proteins being present in the food to use this information for translational purposes.

Taken together, we report for the first time, using a mouse model, that the thaumatin-like protein, Pru av 2, is a potential cherry allergen that causes percutaneous sensitization. The mechanism of Pru av 2 -induced percutaneous sensitization remains to be resolved.

## Figures and Tables

**Table 1:** Amino acid yield of Edman degradation. (Yield(pmol)).

Cycles	1	2	3	4	5	6	7	8
Asp	1.10	5.01	11.94	20.52	24.42	33.92	50.18	56.75
Glu	1.32	5.37	7.45	19.44	27.47	33.45	33.73	34.11
Asn	1.21	2.51	4.28	6.44	8.85	22.27	**258.58 **	**346.43 **
Gln	6.31	6.35	11.15	33.78	30.37	36.25	41.57	45.86
Ser	1.30	2.48	6.02	**142.57 **	49.54	23.91	19.28	20.20
Thr	2.92	**279.90 **	59.23	26.13	28.82	34.38	34.00	37.62
His	0.25	14.61	5.10	2.59	2.60	3.39	4.12	4.25
Gly	16.28	10.38	21.03	31.10	29.91	35.57	39.70	46.69
Ala	**526.88 **	91.78	27.39	42.33	43.13	52.08	57.41	68.76
Tyr	4.70	5.72	5.05	7.92	13.42	17.06	18.24	20.99
Arg	1.44	2.27	2.58	23.59	8.40	6.50	6.89	6.45
Met	0.17	0.16	0.27	0.57	0.80	2.77	3.28	3.69
Val	1.76	3.03	11.25	22.41	28.31	38.54	42.67	45.15
Pro	1.18	9.73	29.07	46.34	56.17	69.89	77.27	86.14
Trp	1.23	0.71	0.73	0.48	2.00	1.07	2.55	2.17
Phe	0.36	4.26	14.78	22.84	**392.79 **	182.05	85.35	66.37
Lys	0.05	0.93	1.73	3.29	8.57	**353.62 **	151.10	70.93
Ile	1.85	6.72	**491.51 **	118.35	22.74	12.88	9.12	14.75
Leu	0.50	5.85	19.65	21.70	21.72	32.54	32.84	38.84

## Data Availability

The data presented in this study are available on request from the corresponding author.
